# A Five-Year Experience of Carbapenem Resistance in Enterobacteriaceae Causing Neonatal Septicaemia: Predominance of NDM-1

**DOI:** 10.1371/journal.pone.0112101

**Published:** 2014-11-18

**Authors:** Saswati Datta, Subhasree Roy, Somdatta Chatterjee, Anindya Saha, Barsha Sen, Titir Pal, Tapas Som, Sulagna Basu

**Affiliations:** 1 Division of Bacteriology, National Institute of Cholera and Enteric Diseases, Kolkata, West Bengal, India; 2 Department of Neonatology, Institute of Postgraduate Medical Education & Research, SSKM Hospital, Kolkata, West Bengal, India; 3 AbsolutData Research and Analytics, Gurgaon, Haryana, India; University of Malaya, Malaysia

## Abstract

Treatment of neonatal sepsis has become a challenge with the emergence of carbapenemase-producing bacteria. This study documents the trend of carbapenem susceptibility in Enterobacteriaceae that caused septicaemia in neonates over a five year period (2007–2011) and the molecular characterisation of Enterobacteriaceae resistant to carbapenems and cephalosporins. Hundred and five Enterobacteriaceae including *Escherichia coli* (n = 27), *Klebsiella pneumoniae* (n = 68) and *Enterobacter spp.* (n = 10) were isolated from blood of septicaemic neonates followed by antibiotic susceptibility tests, determination of MIC values, phenotypic and genotypic detection of β-lactamases. Carbapenem was the most active antimicrobial tested after tigecycline. CTX-M type was the most prevalent ESBL throughout the period (82%). New Delhi Metallo-β-lactamase-1 (NDM-1), which is a recent addition to the carbapenemase list, was the only carbapenemase identified in our setting. Fourteen percent of the isolates possessed *bla*_NDM-1_. Carbapenem non-susceptibility was first observed in 2007 and it was due to loss of Omp F/Ompk36 in combination with the presence of ESBLs/AmpCs. NDM-1 first emerged in *E. coli* during 2008; later in 2010, the resistance was detected in *K. pneumoniae* and *E. cloacae* isolates. NDM-1-producing isolates were resistant to other broad-spectrum antibiotics and possessed ESBLs, AmpCs, 16S-rRNA methylases, AAC(6′)-Ib-cr, bleomycin resistant gene and class 1 integron. Pulsed field gel electrophoresis of the NDM-1-producing isolates indicated that the isolates were clonally diverse. The study also showed that there was a significantly higher incidence of sepsis caused by NDM-1-harbouring isolates in the male sex, in neonates with low birth weight and neonates born at an extramural centre. However, sepsis with NDM-1-harbouring isolates did not result in a higher mortality rate. The study is the first to review the carbapenem resistance patterns in neonatal sepsis over an extended period of time. The study highlights the persistence of ESBLs (CTX-Ms) and the emergence of NDM-1 in Enterobacteriaceae in the unit.

## Introduction

Treatment of neonatal sepsis is a challenge. The treatment needs to be rapid, appropriate for the pathogen and safe for the neonate. The challenge seems to be increasing with each passing day due the escalating multidrug-resistant organisms [1]. In practice, ampicillin or amoxicillin along with an aminoglycoside (amikacin or gentamicin) is the common antibiotic regimen for neonatal sepsis. In case of severe infection due to multidrug-resistant members of the Enterobacteriaceae, including those with extended-spectrum β-lactamases (ESBLs) or AmpCs, carbapenems and quinolones are used as the last resort for treatment [2]. However, with the emergence of carbapenem-resistant isolates this treatment regimen is now under threat.

Carbapenem resistance may occur due to expression of ESBL/AmpC-type enzymes combined with the decreased cellular penetration of carbapenems caused by loss of outer membrane protein. Isolates with this mechanism of resistance often express variable susceptibility to the different carbapenem agents. However, isolates with carbapenemase-mediated resistance are of special clinical concern because multi-institutional outbreaks have been reported worldwide [3].

Carbapenemases are enzymes that not only hydrolyse carbapenems but almost all hydrolysable β-lactams, and most are resistant against inhibition by the β-lactamase inhibitors [4]. Carbapenemase-producing Enterobacteriaceae remained extremely rare for around 20 years after imipenem’s introduction but recently, have begun to accumulate in the Enterobacteriaceae. In particular, *Klebsiella pnemoniae* carbapenemase (KPC, a class A carbapenemase), VIM (class B or metallo-carbapenemase) and OXA-48 (class D carbapenemase) [4] and recently the NDM-1 (metallo-carbapenemase) is widespread in Enterobacteriaceae throughout the world [5].

The New Delhi Metallo-β-lactamase-1 (NDM-1) is the most recent addition to the list of carbapenemases. It is a zinc–requiring metallo–β–lactamase (MBL) that can hydrolyse all penicillins, cephalosporins, carbapenems and spares only the monobactam aztreonam [6]. NDM-1 is often associated with other antibiotic resistance genes and plasmids carrying *bla*_NDM-1_, can have up to 14 other antibiotic resistance determinants and can easily transfer this resistance to other bacteria [7].

This study was carried out in a neonatal intensive care unit (NICU) in which carbapenem resistance in Enterobacteriaceae was rare before 2008. Resistance to carbapenems was more a problem with lactose nonfermenting bacteria like *Acinetobacter baumannii* in the same unit [8], but not in Enterobacteriaceae. However, with the emergence of carbapenem resistance in Enterobacteriaceae it was necessary to evaluate the carbapenem susceptibility patterns in the NICU and the genetic determinants responsible for the resistance. This study focuses on (i) the trend of carbapenem susceptibility in Enterobacteriaceae causing septicaemia in neonates, over a five year period (includes period before and after the emergence of carbapenem resistance) and (ii) the molecular characterisation of carbapenem-resistant and cephalosporin-resistant genes in Enterobacteriaceae isolated during that period. The study is the first to evaluate the carbapenem resistance patterns in neonatal sepsis over an extended period of time.

## Materials and Methods

### Ethics Statement

The study protocol was carefully reviewed and approved by the Institutional Ethics Committee of the National Institute of Cholera and Enteric Diseases (Indian Council of Medical Research) (No. C-48/2010 T & E and NO. C-48/2011- T & E respectively). Individual informed consent was waived because this study used currently existing sample collected during the course of routine diagnosis of sepsis and did not pose any additional risks to the patients. The patient records/information was anonymized and de-identified prior to analysis.

### Setting and patients

The study was conducted at a 20-bed level III unit of the IPGMER and SSKM Hospital, Kolkata, India between 2007 and 2011. The unit is the only Level III unit in the state. This unit has about 1000 admissions per year (departmental census 2010), including both intramural and extramural births.

### Bacterial strains

During 2007–2011, a total of 1985 blood specimens had been drawn from the admitted neonates suspected for sepsis on the basis of criteria set earlier by authors [9], and blood culture procedures followed were as described previously [10]. Of the specimens cultured, 285 were positive (including gram-positive bacteria, gram-negative bacteria and fungal isolates). The clinical data were noted from the hospital registers.

### Laboratory procedures

All Enterobacteriaceae isolated were identified by the ID 32 E kit (bioMérieux, Marcy l’É toile, France). Antibiotic susceptibility profiles and minimum inhibitory concentrations (MIC) were evaluated along with phenotypic tests for the detection of β-lactamases and carbapenemases. Detailed molecular characterization and outer membrane permeability were carried out for the ertapenem-non-susceptible isolates. Molecular typing was performed only for carbapenemase-producing (more specifically NDM-1-producing) isolates.

### Antimicrobial susceptibility and MIC

Antimicrobial susceptibility testing was done by the Kirby-Bauer standard disk diffusion method [11] according to CLSI guidelines [12] for different antimicrobial agents like: ceftazidime (30 µg), cefotaxime (30 µg), cefpodoxime (10 µg), ceftriaxone (30 µg), cefepime (30 µg), aztreonam (30 µg), ampicillin (10 µg), piperacillin (100 µg), cefoxitin (30 µg), gentamicin (120 µg), amikacin (30 µg), ciprofloxacin (5 µg), tetracycline (30 µg), minocycline (30 µg), chloramphenicol (30 µg), trimethoprim/sulfamethoxazole (1.25 µg/23.75 µg), colistin (10 µg), ertapenem (10 µg) and meropenem (10 µg) (BD Diagnostics, Franklin Lakes, NJ, USA).

The MIC values (mg/L) of cefotaxime, ertapenem, meropenem, amikacin, gentamicin and tigecycline were determined using Etest method (AB Biodisk, Solna, Sweden) and were interpreted according to CLSI guidelines as modified in 2013. The clinical breakpoints for meropenem were as follows: susceptible (S) ≤1.0 mg/L, intermediate (I) 2.0–3.0 mg/L, and resistant (R) ≥4.0 mg/L. The same for ertapenem were as follows: S ≤0.5 mg/L, I: 1.0 mg/L, R ≥2 mg/L. MIC50 and MIC90 of meropenem were calculated as the MIC at which 50% and 90% of the isolates were inhibited.

### Screening for ESBLs, AmpCs and Carbapenemases

For all Enterobacteriaceae, the MIC value for ertapenem ≥0.5 mg/L was set as the screening breakpoint to detect carbapenemases [13]. The presence of ESBL was determined according to CLSI guidelines. The AmpC screening breakpoint was set as zone diameter of ≤18 mm for cefoxitin (30 µg) disc [14].

### Detection of β-lactamase and carbapenemase phenotypes

The production of ESBLs, AmpCs, KPC and MBLs were evaluated using cephalosporin/clavulanic acid (BD Diagnostics, Franklin Lakes, NJ, USA) combination disc, cefoxitin (30 µg)/boronic acid (300 µg) (Sigma-Aldrich, St Louis, MO, USA) combination disc [14], meropenem (10 µg)/boronic acid (300 µg) combination disc and imipenem (10 µg)/EDTA (750 µg) (Sigma-Aldrich, St Louis, MO, USA) combination disc test [15] respectively. Isolates exhibiting an increase of ≥5 mm in the inhibition zone of the combination disc were categorized as positive.

### Molecular characterization of β-lactamases, carbapenemases, 16S rRNA methylases and integrons

On the basis of results of the phenotypic tests, PCR was carried out for presence of carbapenemase genes (*bla*_VIM,IMP,SPM-1,GIM-1,SIM-1,KPC,SME,SPM,NDM,GES_) [16]–[19], β-lactamase genes (*bla*_SHV,TEM,OXA-1,CTX-M_) [20], [21], and AmpC genes (*bla*_MOX, CMY, DHA, ACC, MIR/ACT, FOX_) [22]. For ertapenem-non-susceptible isolates, all amplified β-lactamase products were further sequenced on both DNA strands in an automated DNA sequencer (Applied Biosystems 3730, DNA Analyzer, Perkin Elmer, USA) and aligned with the gene sequences available from Genbank (http://www.ncbi.nlm.nih.gov/genbank).

For isolates resistant to either aminoglycoside, genotypic detection for 16S rRNA methylase-encoding genes (*rmtA, rmtB, rmtC, rmtD* & *armA*) were done [23]. Investigation of integron classes (*IntI1, IntI2* and *IntI3* genes) were carried out for all 105 isolates [24].

In case of NDM-1 producing isolates only, association of plasmid-mediated quinolone resistance gene, *aac(6′)-Ib-cr* and bleomycin resistant gene, *ble*_MBL_ were also investigated in addition to other genes listed above [25]. Amplified *ble*_MBL_ gene was further sequenced on both DNA strands to confirm its position with respect to *bla*_NDM-1_.

### Outer membrane permeability

Whole-cell extracts of the ertapenem-non-susceptible Enterobacteriaceae isolates were separated on 11% SDS–polyacrylamide gels [16], and were transferred to Immobilon-P membrane (Millipore) following standard procedures. From our collection, an isolate of *E. coli* (S205) (resistant to all generations of cephalosporins, aminoglycosides, carbapenems, fluoroquinolones and only susceptible to minocycline and colistin) which retained both the porins (Omp C/F) has been used as a control for the western blots. Porins were detected using polyclonal anti-OmpC/F antibody as described earlier [10].

### Pulsed field gel electrophoresis (PFGE) of NDM-1-producing Enterobacteriaceae

PFGE was carried out for all NDM-1-possessing isolates by following PulseNet standardized procedures (http://www.cdc.gov/pulsenet/protocols.htm) in a CHEF-DR III apparatus (Bio-Rad Laboratories, Hercules and CA). XbaI macrorestriction patterns were compared and interpreted according to the criteria of Tenover *et al.* [26]. The dendrogram was generated by FPQuest software, version 4.5 (BioRad Laboratories, Hercules, CA, USA).

### Statistical analysis

Data generated for the above samples and tests were analyzed systematically using established statistical procedures. All data analysis and statistics was done using R version 3.1.1. Association of clinical factors with sepsis caused by NDM-1-harbouring bacteria was evaluated by a multivariate logistic regression. All available clinical factors were entered into the regression at the same time and a backward selection process was used to identify the clinical factors with a significant association with neonates having sepsis due to NDM-1-carrying Enterobacteriaceae. P-values <0.05 were considered statistically significant. The association of the presence of NDM-1-producing bacteria with mortality was tested using a Chi-square test.

## Results and Discussion

### Bacterial isolates

During 2007–2011, 37% of the 285 culture positive isolates yielded Enterobacteriaceae. The 105 non-duplicate clinical isolates of Enterobaceriaceae including *Escherichia coli* (n = 27, 26%), *Klebsiella pneumoniae* (n = 68, 65%), *Enterobacter cloacae* (n = 8, 7.6%) and one each of *Enterobacter amnigenus* and *Enterobacter sakazakii* (0.95%) were analyzed.

### Distribution of MIC values of different groups of antibiotics with focus on carbapenems

Tigecycline was the most active antimicrobial tested against *E. coli*, with 100% susceptibility followed by carbapenems (74% for meropenem and 67% for ertapenem), over the five year period. All other broad-spectrum agents had susceptibility rates ranging between 22% and 74% (22% for cefotaxime, 41% for gentamicin and 74% for amikacin) (Table 1). The resistance to carbapenems in *E. coli* first emerged in 2008 (11% for meropenem, 22% for ertapenem) and the resistance was highest in 2011 (37.5% for meropenem and ertapenem both).

**Table 1:** Antimicrobial activity of meropenem and 5 broad-spectrum comparator agents tested against Enterobacteriaceae during the study period (2007–2011).

Organism (no. tested)/antimicrobial agent	% susceptible^a^						MIC (mg/L)		
	5 years	2007	2008	2009	2010	2011	50%	90%	Range
***Escherichia coli*** (27)		(4)	(9)	(2)	(4)	(8)			
Meropenem	74	100	88.89	50	75	62.5	0.094	32	0.016–≥32
Ertapenem	67	100	77.78	50	75	62.5	0.064	≥32	0.004–≥32
Cefotaxime	22	50	0	0	0	50	32	≥256	0.006–≥256
Amikacin	74	100	88.89	50	50	62.5	8	≥256	2–≥256
Gentamicin	41	50	44.45	50	0	50	32	≥1024	0.125–≥1024
Tigecycline	100	100	100	100	100	100	0.094	0.38	0.047–0.5
***Klebsiella pneumoniae*** (68)		(20)	(12)	(10)	(15)	(11)			
Meropenem	91	100	100	100	66.7	91	0.094	2	0.047–32
Ertapenem	87	100	100	70	66.7	91	0.125	12	0.012–≥32
Cefotaxime	9	15	8.33	10	0	9	32	≥256	0.032–≥256
Amikacin	56	50	66.7	100	20	63.6	12	≥256	1.5–≥256
Gentamicin	19	25	0	30	26.7	9	96	≥1024	0.38–≥1024
Tigecycline	96	100	91.7	90	100	91	0.5	1.5	0.19–8

a the susceptibility was determined according to the CLSI-2013 MIC interpretative criteria.

In case of *K. pneumoniae*, tigecycline was again the most active antimicrobial with 96% susceptibility, closely followed by carbapenems (91% for meropenem and 87% for ertapenem). All other broad-spectrum agents had susceptibility rates ranging between 9% and 56% (9% for cefotaxime, 19% for gentamicin and 56% for amikacin). (Table 1). The resistance to carbapenems in *K. pneumoniae* isolates did not appear until 2009 (30% for ertapenem) and the resistance to meropenem emerged in 2010. The resistance to carbapenems was highest in 2010 (33% for meropenem and ertapenem both). In contrast, in 2011, there was only 9% resistance towards carbapenems indicating decreasing resistance rates.

A comparison of the susceptibility profiles of the two organisms revealed that susceptibility rates for cefotaxime, gentamicin and amikacin among *K. pneumoniae* isolates are lower than *E. coli* isolates. But with respect to carbapenems, susceptibility rates for *K. pneumoniae* isolates are higher than *E. coli* isolates during the study period (Table 1). However, with very few *E. coli* isolates these differences should not be overemphasized.

For *E. cloacae* (n = 8), the highest resistance was observed with cefotaxime (100%) followed by amikacin and gentamicin (87.5%), ertapenem (50%) and meropenem (37.5%). Tigecycline was the only agent for which 100% susceptibility was found. The resistance to ertapenem in *E. cloacae* isolates emerged in 2008 (n = 1) but resistance to meropenem was observed in late 2010 (n = 1); and was highest during 2011 (n = 2). Due to small number of *Enterobacter* isolates, percentage calculation as well as MIC50, MIC90 determination was not carried out. *Enterobacter amnigenus* and *Enterobacter sakazakii* were susceptible to all antimicrobial agents tested in this study.

**Figure 1:**
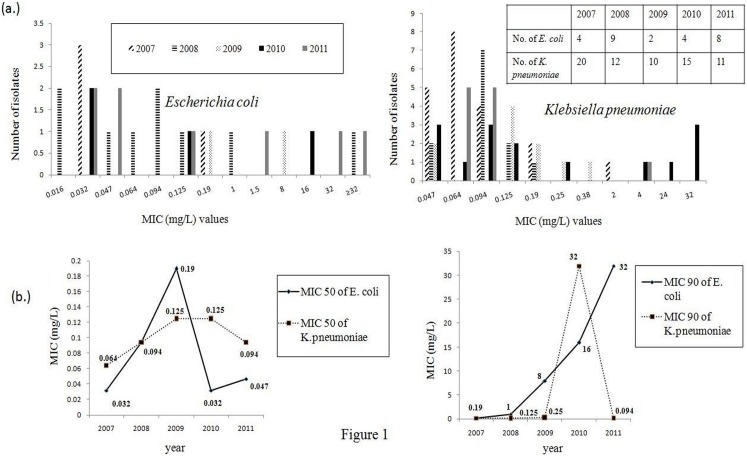
Meropenem MIC values : (a) Distribution of Meropenem MIC values among 27 *E*. *Coli* isolates and 68 *K. pneumoniae* isolates as determined by the Etest method during the study period (2007–2011); (b) Graphical representation of MIC50 and MIC90 values of the isolates throughout five year period.

The MIC values inhibiting 50% (MIC50) and 90% (MIC90) of the organisms tested against cefotaxime, ertapenem, meropenem, amikacin, gentamicin and tigecycline are presented in Table 1. As this study focuses on carbapenem resistance, detailed distribution of meropenem MIC values of *E. coli* and *K. pneumoniae* isolates for each year has been depicted in Figure 1a and 1b, respectively.

The distribution of the MIC90 values indicated an upward shift of meropenem MICs among the *E. coli* isolates from 2007 to 2011. Since for *E. coli*, the sample number is small, particularly for the numbers in an individual year, the MIC50 and MIC90 obtained in this study should not be overstated as a few strains with high MICs may skew the result. For *K. pneumoniae* isolates, the range of MIC values and MIC90 values indicated an abrupt upward shift from 2009 to 2010, which did not persist in the following year (2011).

MIC50 values for both *E. coli* and *K. pneumoniae* isolates remained within the susceptible range based on the CLSI guidelines as modified in 2013. In addition, a comparison of the two organisms revealed that the number of *E. coli* isolates non-susceptible to meropenem increased constantly throughout the study period while among *K. pneumoniae,* non-susceptible isolates were first detected in 2010 but the number of such isolates declined in 2011.

As the MIC value for ertapenem ≥0.5 mg/L was selected as the screening breakpoint for carbapenemases, a comparison of the susceptibilities to other antibiotics (cefotaxime, amikacin, gentamicin and tigecycline) among ertapenem-susceptible and ertapenem-non-susceptible isolates (Table S1) was carried out. This showed that ertapenem non-susceptible isolates had decreased susceptibilities to other antibiotics. This result supports other earlier studies from different countries where carbapenem-non-susceptible isolates show reduced susceptibility to other classes of antibiotics [27], [28].

### Detection of β–lactamases based on phenotypic tests

Ten Enterobacteriaceae isolates (4 *E. coli,* 4 *K. pneumoniae*, each of *Enterobacter amnigenus* and *Enterobacter sakazakii*) were susceptible to all generations of cephalosporins, monobactam and carbapenems as tested by the disc diffusion test. The remaining Enterobacteriaceae (n = 95) were further analysed for production of β-lactamases by phenotypic tests (Figure S1). Seventy six percent (n = 80) and 8.5% (n = 9) were detected as ESBL- and AmpC-producers, respectively.

Twenty-six isolates with elevated ertapenem MICs (≥0.5 mg/L) were considered for KPC and MBL analysis. None were positive for KPC production but fifteen isolates (6 *E. coli*, 6 *K. pneumoniae* and 3 *E. cloacae*) produced MBLs. These fifteen isolates were also non-susceptible to meropenem (>1 mg/L). MBL-producing bacteria showed inconclusive phenotypic results for ESBLs and AmpCs. Therefore, the presence of ESBLs and AmpCs in these isolates was confirmed by PCR subsequently (Figure S1).

The phenotypic detection of ESBLs and AmpCs in presence of MBLs is challenging, indicating that further development of phenotypic tests for ESBL detection in MBL–producing isolates is of utmost importance. The failure to detect the ESBLs in the MBL–producing clinical isolates may lead to the hidden spread of such β- lactamases complicating the situation even further.

### Genotypic distribution of β-lactamases among Enterobacteriaceae

Isolates categorized as positive by the different phenotypic tests were further analysed for the cephalosporin-resistant and carbapenem-resistant genes. Isolates harboured different combinations of any of the three ESBL types (CTX-M, SHV and TEM), two AmpC types (CMY and ACT) and only one carbapenemase type (NDM) (Table S2). The most common β-lactamase was CTX-M group 1, present in 82% of the isolates (n = 86), followed by TEM in 70% (n = 74) and SHV in 45% of the isolates (n = 47). The most common AmpC β-lactamase type was CMY (n = 5), followed by ACT (n = 2). The yearwise breakup of the ESBLs, AmpCs and carbapenemases are presented in Table 2. There were very few isolates that did not possess any of these genes.

**Table 2:** Distribution of resistance determinants among Enterobacteriaceae isolates (2007–2011).

Isolate and resistance determinants	Total (%)	2007	2008	2009	2010	2011
		No. of strains				
***Escherichia coli***	27 (26)	4	9	2	4	8
*bla*_SHV_	6 (26)	0	3	0	1	2
*bla*_TEM_	16 (69)	0	7	1	3	5
*bla*_OXA_	8 (35)	2	2	1	0	3
*bla*_CTXM_	20 (87)	2	6	1	4	7
*bla*_CMY_	5 (19)	0	2	1	0	2
*bla*_NDM_	**6 (22)**	**0**	**1**	**1**	**1**	**3**
Negative for all determinants	4 (15)	2	2	0	0	0
***Klebsiella pneumoniae***	68 (65)	20	12	10	15	11
*bla*_SHV_	40 (63)	14	2	10	10	4
*bla*_TEM_	51 (80)	17	11	8	11	4
*bla*_OXA_	30 (47)	11	6	3	5	5
*bla*_CTXM_	58 (91)	17	9	8	14	10
*bla*_NDM_	**6 (9)**	**0**	**0**	**0**	**5**	**1**
Negative for all determinants	4 (6)	2	1	0	0	1
***Enterobacter cloacae***	8 (7.6)	1	2	1	3	2
*bla*_SHV_	1 (12)	0	0	0	1	0
*bla*_TEM_	7 (87)	1	1	1	2	2
*bla*_OXA_	6 (75)	1	1	1	2	1
*bla*_CTXM_	8 (100)	1	1	1	3	2
*bla*_ACT_	2 (25)	0	1	0	1	0
*bla*_NDM_	**3 (37)**	**0**	**0**	**0**	**1**	**2**
Negative for all determinants	0 (0)	0	0	0	0	0

Sequencing for all the genes on both strands have been carried out in the ertapenem-non-susceptible isolates. Table 3 and Table 4 demonstrates the distribution of β-lactamases in these isolates.

**Table 3:** Microbiological and molecular characterization of non-NDM-harbouring Enterobacteriaceae isolates with ertapenem MIC ≥0.5 mg/L.

Isolate no.	Period of isolation	organism	MIC Values (mg/L)								Genetic determinants	Integrons	Porins
			**CT**	**ETP**	**MP**	**AK**	**GM**	**CI**	**TGC**	**CL**			
I1	Mar, 2007	*Klebsiella pneumoniae*	>256	0.5	0.094	8	96	3	ND#	ND	*bla*_SHV-28,_ *bla*_TEM-1,_ *bla*_OXA-1,_ *bla*_CTX-M-28_		Ompk35, Omp A
I2	Aug, 2007	*Klebsiella pneumoniae*	>256	0.5	0.094	24	13	4	ND	ND	*bla*_SHV-61,_ *bla*_TEM-1,_ *bla*_OXA-1,_ *bla*_CTXM-22_	IntI1	Ompk35, Omp A
I3	Aug, 2007	*Escherichia coli*	>256	0.5	0.19	16	128	>32	ND	ND	*bla*_OXA-1,_ *bla*_CTXM-28,_ *rmt B*		Omp C, Omp A
I4	Sep, 2007	*Enterobacter cloacae*	>256	0.5	0.19	≥256	≥1024	>32	ND	ND	*bla*_TEM-1,_ *bla*_OXA-1,_ *bla*_CTXM-15_		Omp C, Omp A
I5	Jan, 2008	*Enterobacter cloacae*	>256	24	0.25	16	≥1024	>32	0.125	0.25	*bla*_TEM-1,_ *bla*_OXA-1,_ *bla*_CTXM-15,_ *bla*_ACT-7_		Omp C, Omp A
I6	Sep, 2008	*Klebsiella pneumoniae*	>256	0.5	0.047	12	162	>32	0.5	0.16	*bla*_TEM-1,_ *bla*_OXA-1,_ *bla*_CTXM-15_	IntI1	Ompk35, Omp A
I7	Dec, 2008	*Escherichia coli*	>256	>32	1	≥256	≥1024	>32	0.064	0.125	*bla*_TEM-1,_ *bla*_OXA-1,_ *bla*_CTXM-15,_ *bla*_CMY-4_	IntI1	Omp C, Omp A
I8	Jan, 2009	*Klebsiella pneumoniae*	>256	2	0.19	8	48	>32	8	1	*bla*_SHV-11,_ *bla*_TEM-1,_ *bla*_OXA-1,_ *bla*_CTXM-15_	IntI1	Ompk35, Omp A
I9	Jul, 2009	*Klebsiella pneumoniae*	>256	32	0.125	8	128	>32	1	0.38	*bla*_SHV-1,_ *bla*_TEM-1,_ *bla*_OXA-1,_ *bla*_CTXM-15_	IntI1	Ompk35, Omp A
I10	Aug, 2009	*Klebsiella pneumoniae*	>256	1	0.125	2	>1024	>32	1	0.25	*bla*_SHV-1,_ *bla*_TEM-1,_ *bla*_OXA-1,_ *bla*_CTXM-15_		Ompk35, Omp A
I11	Dec, 2009	*Klebsiella pneumoniae*	>256	0.5	0.38	2	0.75	0.75	0.75	0.5	*bla*_SHV-1_	IntI1	Ompk35, Omp A

CT: Cefotaxime, ETP: Ertapenem, MP: Meropenem, AK: Amikacin, GM: Gentamicin, CI: Ciprofloxacin, TGC: Tigecycline, CL: Colistin; IntI1: class 1 integron; Omp: Outer membrane protein.

# ND: Not Determined.

**Table 4:** Antibiotic susceptibility and molecular characterization of NDM-1- harbouring Enterobacteriaceae along with clinical features of the neonates harbouring the same isolates in their blood specimens.

Patient no./Organism(isolate no.)	Sex	Inborn Or Outborn	Birth weight	Gestational Age$	Mode Of delivery	ventilation	Prescribed antibiotics	Outcome	MIC Values (mg/L)								Genetic determinants	Integron	Porins
									CT	ETP	MP	AK	GM	CI	TGC	CL			
P1/*Escherichia coli* (E1)	M	Inborn	LBW	preterm	LUCS	No	PipTaz/Amika	discharge	>256	>32	>32	>256	>1024	>32	0.25	1	*bla*_TEM-1,_ *bla*_CTXM_-_15_, *bla*_CMY-6_, *rmt C, aac(6′)-Ib, ble*_MBL_	IntI1	Omp C, Omp A
P2/*Escherichia coli* (E2)	M	Inborn	VLBW	preterm	LUCS	Yes	PipTaz/Amika	discharge	>256	>32	8	>256	>1024	>32	0.25	0.5	*bla*_TEM-1_, *bla*_OXA-1_, *bla*_CMY-42_, *arm A, aac(6′)-Ib-cr, ble*_MBL_		Omp C, Omp F, Omp A
P3/*Klebsiella pneumoniae* (K1)	M	Outborn	NW	term	NVD	Yes	colistin	discharge	>256	>32	32	>256	>1024	>32	0.75	0.25	*bla*_SHV-11_, *aac(6′)-Ib*, *ble*_MBL_	IntI1	Ompk35, Omp A
P4/*Klebsiella pneumoniae* (K2)	M	Outborn	LBW	preterm	LUCS	No	ofloxacin	discharge	>256	>32	24	>256	>1024	3	1.5	0.38	*bla*_SHV-167_, *bla*_CTXM_-_15_, *arm A, aac(6′)-Ib-cr*, *ble*_MBL_	IntI1	Ompk35, Omp A
P5/*Klebsiella pneumoniae* (K3)	M	Outborn	LBW	preterm	NVD	Yes	ofloxacin	discharge	>256	12	4	>256	>1024	>32	1	0.75	*bla*_TEM-1_, *bla*_OXA-1_, *bla*_CTXM_-_15_, *aac(6′)-Ib*, *ble*_MBL_	IntI1	Ompk35, Omp A
P6/*Klebsiella pneumoniae* (K4)	M	Outborn	LBW	term	LUCS	Yes	colistin	discharge	>256	>32	32	>256	>1024	>32	1	0.38	*bla*_TEM-1_, *bla*_OXA-1_, *bla*_CTXM_-_15_, *aac(6′)-Ib, ble*_MBL_	IntI1	Ompk35, Omp A
P7/*Escherichia coli* (E3)	M	Inborn	LBW	preterm	NVD	No	meropenem	death	>256	>32	16	>256	>1024	>32	0.25	0.38	*bla*_TEM-1_, *bla*_CTXM_-_15_, *rmt B, ble*_MBL_	IntI1	Omp C, Omp F, Omp A
P8/*Klebsiella pneumoniae* (K5)	F	Outborn	VLBW	preterm	LUCS	Yes	meropenem	discharge	>256	>32	32	>256	>1024	>32	0.38	0.75	*bla*_SHV-11_, *bla*_OXA-1_, *bla*_CTXM_-_15_, *aac(6′)-Ib, ble*_MBL_	IntI1	Ompk35, Omp A
P9/*Enterobacter cloacae* (EC1)	M	Outborn	LBW	preterm	NVD	Yes	colistin/ofloxacin	LAMA	>256	>32	>32	>256	>1024	4	0.25	1.5	*bla*_OXA-1_, *bla*_CTXM_-_15_, *bla*_ACT-16_, *rmt C, aac(6′)-Ib, ble*_MBL_	IntI1	Omp C, Omp A
P10/*Enterobacter cloacae* (EC2)*	M	Outborn	VLBW	preterm	NVD	Yes	colistin	discharge	>256	8	6	>256	>1024	2	0.5	0.5	*bla*_TEM-1_, *bla*_OXA-1_, *bla*_CTXM_-_15_, *rmt B, aac(6′)-Ib-cr, ble*_MBL_	IntI1	Omp C, Omp A
P10/*Escherichia coli* (E4)*	M	Outborn	VLBW	preterm	NVD	Yes	colistin	discharge	>256	>32	>32	>256	>1024	>32	0.125	0.75	*bla*_TEM-1_, *bla*_CTXM_-_15_, *bla*_CMY-42_, *rmt B, ble*_MBL_	IntI1	Omp C, Omp F, Omp A
P11/*Escherichia coli* (E5)	M	Inborn	LBW	term	LUCS	No	ofloxacin	discharge	>256	>32	32	>256	>1024	>32	0.38	1.5	*bla*_CTXM_-_15_, *rmt B, aac(6′)-Ib-cr, ble*_MBL_	IntI1	Omp C, Omp F, Omp A
P12/*Klebsiella pneumoniae* (K6)**	No clinical data available								>256	12	4	>256	>1024	8	0.75	1	*bla*_TEM-1,_ *bla*_OXA-1_, *bla*_CTXM_-_15_,*rmt B,* *aac(6′)-Ib, ble*_MBL_	IntI1	Ompk35,Omp A
P13/*Enterobacter cloacae* (EC3)	M	Outborn	VLBW	preterm	NVD	Yes	PipTaz/Amika	death	>256	>32	32	>256	>1024	8	1.5	1	*bla*_TEM-1_, *bla*_CTXM_-_15_, *aac(6′)-Ib, ble*_MBL_	IntI1	Omp C, Omp A
P14/*Escherichia coli* (E6)	M	Outborn	VLBW	preterm	NVD	No	ofloxacin	discharge	>256	>32	1.5	>256	>1024	>32	0.094	0.75	*bla*_TEM-1_, *bla*_OXA-1_, *bla*_CTXM_-_15_, *bla*_CMY-42,_ *rmt B, aac(6′)-Ib*	IntI1	Omp C, Omp F, Omp A

M: male; F: female; LBW: low birth weight (<2500 gm); VLBW: very low birth weight (<1500 gm); NW: normal weight (>2500 gm); NVD: normal vaginal delivery; LUCS: low uterine caesarean delivery; Omp: Outer membrane protein; LAMA: left against medical advice.

CT: Cefotaxime; ETP: Ertapenem; MP: Meropenem; AK: Amikacin; GM: Gentamicin; CI: Ciprofloxacin; TGC: Tigecycline; CL: Colistin; PipTaz: Piperacillin/Tazobactam; Amika: Amikacin;

$ Gestational age <37 weeks is considered as preterm.

*Two different Enterobacteriaceae isolates (1 *E. coli* and 1 *E. cloacae*) were isolated from blood of a single patient.

**No clinical data was available for this patient.

NDM-1 was present in 14% (n = 15) of the isolates and was the only carbapenemase type identified. No other carbapenemases were detected in this study. Class 1 integron was observed in 69 isolates (66%).

Figure S2 depicts the prevalence of ESBLs, AmpCs and NDM-1 over the period of five years. ESBLs have a consistently high prevalence throughout the period while AmpCs have a variable trend after their emergence in 2008. It is noteworthy that NDM-1 has an increasing trend after its emergence in 2008.

We had earlier reported the presence of NDM-1 in *K. pneumoniae* in 2 cases of neonatal septicaemia in 2010 [19]. However, this retrospective study showed that NDM-1 in *E. coli* had actually emerged in 2008 and much later (2010) in *K. pneumoniae*.

### Molecular characterization of ertapenem-non-susceptible isolates which did not produce NDM-1

Eleven (7 *K. pneumoniae*, 2 *E. coli* and 2 *E. cloacae*) out of twenty-six ertapenem-non-susceptible isolates did not produce NDM-1 or any other carbapenemases. Microbiological and molecular characterization of these isolates has been documented in Table 3. All possessed different combinations of ESBLs, particularly CTX-M-15. Two isolates also produced AmpCs (CMY-4 and ACT-7). Evaluation of the porins showed that all of them lack a structural protein, OmpF (in *E. coli*)/OmpK36 (in *K. pneumoniae*). A loss of porin in combination with ESBLs/AmpCs is the reason for carbepenem-resistance in these isolates; these mechanisms of resistance have been documented earlier by other authors also [29].

### Co-existence of multiple resistant-genes along with NDM-1

The molecular characterization of NDM-1-producing Enterobacteriacae (n = 15) is represented in Table 4. Most NDM-1-producing isolates possess multiple β-lactamases, aminoglycoside-resistant genes *armA* or *rmtB* and plasmid mediated quinolone resistant gene *aac(6′)-Ib-cr*. This result indicates that the NDM-1 possessing isolates are associated with unrelated broad-spectrum resistance genes, suggesting that they have been selected by wide range antibiotics. Two novel β-lactamases were also identified in two isolates harbouring NDM-1, a new SHV-type, SHV-167 (GenBank accession no. AB733453) and an AmpC gene, ACT-16 (GenBank accession no. AB737978). The presence of these novel β-lactamases has been reported [30]. Fourteen NDM-1 carrying isolates also possessed *ble*_MBL_ immediately downstream of the *bla*_NDM-1_ gene. This association has been quite systematically identified throughout the world [25].

The fact that class1 integrons were detected in nearly all isolates harbouring NDM-1 makes the situation even more worrisome. Class1 integrons are important players in driving the evolution of complex and laterally mobile multidrug-resistant units [31], [32]. Class 1 integrons have been isolated earlier from NDM-1 harbouring isolates and other multidrug-resistant isolates [33], [34].

The distribution of different classes of resistant determinants among NDM-1-harbouring isolates and isolates not harbouring NDM-1 is described in Table 5. This genetic distribution clearly indicated the association of multiple resistance genes along with NDM-1 gene as has also been reported by other authors [35]. With a wide battery of resistance determinants, NDM-1-possessing isolates remain only suscepltible to colistin and tigecycline. Aztreonam as an alternative also does not stand a chance, as a substantial proportion of the NDM-1 isolates are reported to co-produce CTX-Ms [5]. This study also shows that all NDM-1 isolates possessed CTX-M-15 which is probably widespread in this setting.

**Table 5:** Comparison of the presence of resistance determinants and integrons between NDM-1- producing and non-producing Enterobacteriaceae isolates.

Resistance determinants	NDM-1 isolates (%) (n = 15)	Non-NDM-1 isolates (%) (n = 90)
**ESBL- producer**	15 (100)	80 (89)
*bla*_CTX-M_	13 (87)	73 (81)
*bla*_SHV_	3 (20)	44 (49)
*bla*_TEM_	10 (67)	64 (71)
*bla*_OXA-1_	8 (53)	36 (40)
**AmpC- producer**	5 (33)	2 (2)
*bla*_CMY_	4 (27)	1 (1)
*bla*_ACT_	1 (7)	1 (1)
**16s r-RNA methylase producer**	10 (67)	7 (7)
**Integrons**	14 (93)	64 (71)

ESBL: Extended Spectrum β Lactamase; NDM: New Delhi Metallo-β-lactamase.

### Outer membrane permeability of NDM-1-possessing isolates

The wide range of MIC values (1.5–≥32 mg/L) for meropenem among NDM-1 positive isolates prompted us to examine whether loss of porin was associated with such differences.

All 15 NDM-1-producing isolates retained normal levels of OmpA, a structural protein but OmpF/OmpK36 was not detected in 6 *K*. *pneumoniae* (K1-K6), 3 *E*. *cloacae* (EC1-EC3) and 1 *E. coli* (E1) isolates (Table 4). OmpF/OmpK36 is generally lost or has reduced expression in most ESBL-producing strains. However, loss of porins could not be correlated to differences in MIC values of meropenem. Loss of porin was observed in isolates with MIC values of 4 mg/L, 6 mg/L as well as 24 mg/L, ≥32 mg/L in case of *K. pneumoniae*. All the porins were detected in 5 *E. coli* (E2-E6) isolates with MIC values 1.5 mg/L, 8 mg/L, 16 mg/L, 32 mg/L and ≥32 mg/L. Therefore, loss of porin seemed to be species specific and the differences in MIC values of the NDM-1 possessing isolates probably did not result due to absence of porins. Further work is in progress to understand the reason for such differences which can occur due to alterations in the expression of the enzymes or other changes in the outer membrane proteins.

However, it should be noted that all eleven ertapenem-non-susceptible isolates showed loss of porin which along with the ESBLs was the cause for carbapenem-nonsusceptibility.

### Diversity of the NDM-1 possessing isolates

**Figure 2:**
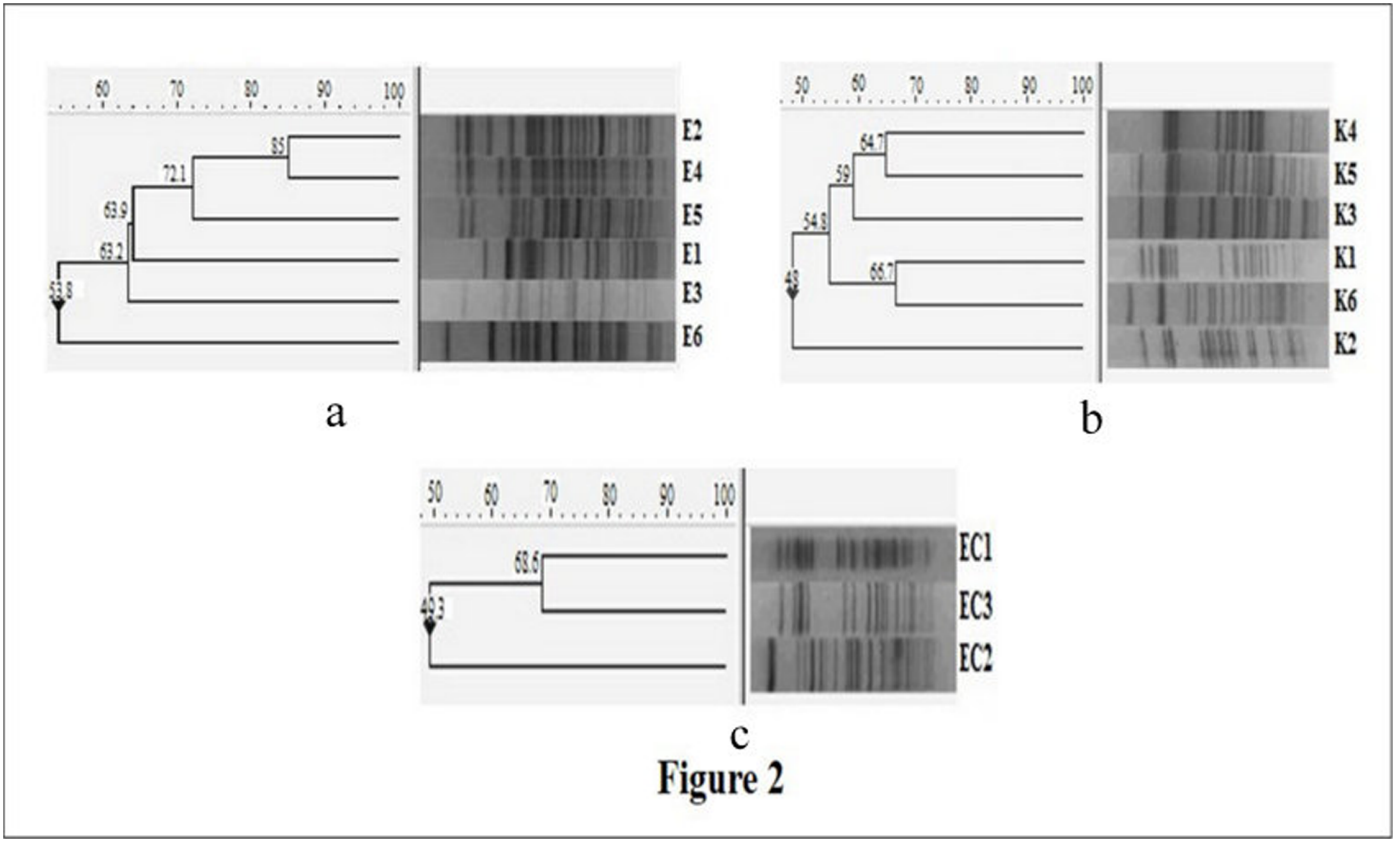
Analysis of the genetic relationship according to Dice’s similarity coefficient and the unweighted pair group method with arithmetic mean (UPGMA) (the position tolerance and optimization were set at 1.0 and 1.0% respectively) of the XbaI patterns of *E. coli* (E1-E6) (a), *K. pneumoniae* (K1-K6) (b) and *E. cloacae* (EC1-EC3) (c). *Salmonella* serotype Braenderup H9812 has been used as reference standard.

PFGE revealed that all NDM-1 carrying Enterobacteriaceae isolates were clonally diverse (Figure 2) and most cases did not cluster in time. No epidemic clone was found to exist during this period. This indirectly indicated the horizontal transmission of carbapenem resistance among these isolates and not cross-transmission among the neonates. However, the fact that most neonates with septicaemia due to NDM-1 possessing Enterobactericeae were referred from other hospitals (Table 4) could also be a reason for the diversity of the clones.

### Clinical presentation of the neonates carrying NDM-1

An evaluation of the demographics and clinical data (Table 4) revealed that most neonates with septicaemia where the causative organism harboured NDM-1 were of low birth weight (n = 7) or very low birth weight (n = 5), preterm (n = 10), ventilated (n = 8), male (n = 12) and born at an extramural centre (outborn) (n = 9). Most of these neonates survived after treatment and were discharged (n = 11).

Cefotaxime along with amikacin or gentamicin was used in the NICU as a pre-emptive antimicrobial therapy for clinically suspected cases of sepsis during 2007–2008. This was changed to piperacillin/tazobactam and amikacin due to the high prevalence of CTX-M gene in the unit. For serious cases of infection, meropenem, ofloxacin and colistin in different combinations were used particularly after the emergence of carbapenem resistance.

### Association of clinical factors and mortality of the neonates with the presence of NDM-1 harbouring-Enterobacteriaceae

The results of a comparison of neonates with presence of NDM-1-producing bacteria in their blood sample and those without NDM-1 are shown in Table 6. Multivariate logistic regression identified significantly higher incidence of sepsis with NDM-1-harbouring Enterobacteriaceae in male neonates as compared to females [Odds ratio (OR) 14.2; p-value 0.01521]. In addition, outborn neonates were also found to have a significantly higher incidence of sepsis due to NDM-1-carrying bacteria[OR 0.19; p-value 0.01877]. Finally, neonates with a low birth weight also had a significantly higher incidence of NDM-1-producing Enterobacteriaceae in their blood sample [OR 9.04; p-value 0.04989]. None of the other clinical factors tested had a significant association with the presence of NDM-1-possessing bacteria.

**Table 6:** Association of clinical factors with sepsis caused by NDM-1 harbouring Enterobacteriaceae in neonates.

Clinical factors		Neonates with NDM-1 harbouring Enterobacteriaceae in Blood (n = 14)$		Neonates without NDM-1 harbouring Enterobacteriaceae in Blood (n = 87)#		P value
No. of Neonates		n	%	n	%	
Sex	Male	13	93	43	49.4	0.01521**
	Female	1	7	44	50.6	
Gestational Age	Pre-term	11	78.6	52	59.8	-
	Term	3	21.4	35	40.2	
Inborn/Outborn	Inborn	4	28.6	59	67.8	0.01877**
	Outborn	10	71.4	28	32.2	
Birth Weight	Low birth weight	13	93	60	69	0.04989**
	Normal birth weight	1	7	26	29.8	
Baby on Ventilation	Yes	9	64.3	36	41.4	-
	No	5	35.7	49	56.3	
Onset of Sepsis*	Early	4	28.6	34	39	-
	Late	9	64.3	52	59.8	
Mode of Delivery	Caesarean delivery	5	35.7	34	39	-
	Normal vaginal delivery	8	57	53	61	

**Significant at 95% confidence.

$ No clinical data was available for one patient.

# No clinical data was available for three patients.

*Early onset of sepsis (<72 hours of birth), Late onset sepsis (>72 hours of birth).

Description of other clinical factors has been described in the footnote of Table 4.

In order to get an indication of the association between the presence of NDM-1-carrying Enterobacteriaceae in blood with mortality, a simple association analysis between the two was conducted. The results showed that neonates with NDM-1-harbouring Enterobacteriaceae in their blood actually had a mortality rate of 13.33% (2 out of 15 neonates). In comparison, the neonates without NDM-1-possessing Enterobacteriaceae in their blood had a mortality rate of 22.22% (20 out of 90 neonates). The difference in the mortality between septicaemic neonates with and without the presence of NDM-1-producing Enterobacteriaceae was not statistically significant [P-value 0.6595].

As there are no previous studies that have analysed the association of clinical factors with sepsis due to NDM-1-carrying organisms in neonates, comparisons with earlier studies is not possible. Comparisons could only be made with studies where risk factors for neonatal sepsis have been investigated. One particular study has shown that male sex is associated with sepsis in neonates [36]. Low birth weight is a risk for sepsis as seen in other studies [37], [38]. Neonates with low birth weight are more vulnerable to infections and thus the association with NDM-1 harbouring Enterobacteriace seems plausible. Neonates born at an extramural centre (outborn) were found to have a significantly higher incidence of sepsis with NDM-1-possessing Enterobacteriacae lending support to the diversity of the isolates as seen by PFGE in this study.

Sepsis caused by NDM-1-producing Enterobateriacae was not associated with mortality of the neonates in this study though one particular study in adults have shown that infections with carbapenem- resistant isolates had a higher mortality rate [39].

## Conclusion

The emergence of carbapenem resistance particularly in Enterobacteriaceae is a considerable burden on the neonatal healthcare system in developing countries. This is the first analysis of the carbapenemases in a neonatal intensive care unit for an extended period of time. The study tries to capture the trend in resistance for a period before and after the emergence of NDM-1 in the unit. The study shows the persistence of the CTX-M-15 gene throughout the five year period. In fact, a prelude to carbapenem resistance, as observed in this study, has been the presence of ESBLs particularly CTX-Ms. Earlier studies from our laboratory and other studies from India have shown the extensive dissemination of this gene [40], [41]. Before the emergence of NDM-1, CTX-M-15 along with porin-loss were the reason for carbapenem-non-susceptibility. However, the prevalence of the AmpC–β- lactamases or the aminoglycoside resistance has shown a rise with the emergence of NDM-1. The association of NDM-1 with other resistance genes has been frequently observed in Enterobacteriacae in other studies also [35].

Though present in the same setting, *K*. *pneumoniae* and *E. coli* displayed differences in susceptibility patterns. *K. pneumoniae* showed higher susceptibility to carbapenems but lower to cefotaxime, gentamicin and amikacin. Such differences in the species has also been noted in other studies [42]. However, with very few *E. coli* isolates, particularly in individual years, these differences should not be overstated.

Though a number of variants of the NDM gene have been reported till date, no variants of this gene were identified in this study. All isolates possessed NDM-1. In addition throughout the five-year period no other MBL was detected in these isolates. The diversity of the isolates indicates probable horizontal transfer of the NDM-1 gene either through plasmids or by the transposons related acquisition. The capability of NDM-1 to associate with other resistance genes raises serious concerns. The other cause for concern is the difficulty in detection of ESBLs in the presence of NDM-1 which hydrolyse carbapenems and also cephalosporins.

The high prevalence of ESBLs and the increasing presence of carbapenemases, both of which can be attributed to horizontal transfer, are indeed worrisome. Added to this is a vulnerable group of critical patients, the hectic environment of the ICU where lapses in infection control may happen and extremely capable pathogens. This is a fatal combination and necessary systemic steps need to be implemented soon.

## Supporting Information

Figure S1 **Schematic representation of the molecular analysis of Enterobacteriaceae isolates enrolled in this study.**
(TIF)

Figure S2 **Graphical representation of percentage of Enterobacteriaceae isolates producing ESBLs, AmpC and NDM during the study period.**
(TIF)

Table S1 **Susceptibility patterns of the ertapenem susceptible and non-susceptible Enterobacteriaceae isolates for 4 broad spectrum** **antibiotics.**
(DOCX)

Table S2 **Microbiological and molecular characterization of 105 Enterobacteriaceae isolates included in this study.**
(DOCX)
