# Correction: A Five-Year Experience of Carbapenem Resistance in Enterobacteriaceae Causing Neonatal Septicaemia: Predominance of NDM-1

**DOI:** 10.1371/journal.pone.0134079

**Published:** 2015-09-01

**Authors:** Saswati Datta, Subhasree Roy, Somdatta Chatterjee, Anindya Saha, Barsha Sen, Titir Pal, Tapas Som, Sulagna Basu

Gene names Ompk36 and Ompk35 are swapped incorrectly throughout the manuscript. All instances of Ompk35 should appear as Ompk36, and all instances of Ompk36 should appear as Ompk35. Corrected versions of Tables 3 and 4, which contain references to Ompk35, can be viewed below with the correct references to Ompk36.

**Table 3:** Microbiological and molecular characterization of non-NDM-harbouring Enterobacteriaceae isolates with ertapenem MIC ≥0.5 mg/L.

Isolate no.	Period of isolation	organism	MIC Values (mg/L)								Genetic determinants	Integrons	Porins
			CT	ETP	MP	AK	GM	CI	TGC	CL			
I1	Mar, 2007	*Klebsiella pneumoniae*	>256	0.5	0.094	8	96	3	ND^#^	ND	*bla*_SHV-28,_ *bla*_TEM-1,_ *bla*_OXA-1,_ *bla*_CTX-M-28_		Ompk36, Omp A
I2	Aug, 2007	*Klebsiella pneumoniae*	>256	0.5	0.094	24	13	4	ND	ND	*bla*_SHV-61,_ *bla*_TEM-1,_ *bla*_OXA-1,_ *bla*_CTXM-22_	IntI1	Ompk36, Omp A
I3	Aug, 2007	*Escherichia coli*	>256	0.5	0.19	16	128	>32	ND	ND	*bla*_OXA-1,_ *bla*_CTXM-28,_ *rmt B*		Omp C, Omp A
I4	Sep, 2007	*Enterobacter cloacae*	>256	0.5	0.19	≥256	≥1024	>32	ND	ND	*bla*_TEM-1,_ *bla*_OXA-1,_ *bla*_CTXM-15_		Omp C, Omp A
I5	Jan, 2008	*Enterobacter cloacae*	>256	24	0.25	16	≥1024	>32	0.125	0.25	*bla*_TEM-1,_ *bla*_OXA-1,_ *bla*_CTXM-15,_ *bla*_ACT-7_		Omp C, Omp A
I6	Sep, 2008	*Klebsiella pneumoniae*	>256	0.5	0.047	12	162	>32	0.5	0.16	*bla*_TEM-1,_ *bla*_OXA-1,_ *bla*_CTXM-15_	IntI1	Ompk36, Omp A
I7	Dec, 2008	*Escherichia coli*	>256	>32	1	≥256	≥1024	>32	0.064	0.125	*bla*_TEM-1,_ *bla*_OXA-1,_ *bla*_CTXM-15,_ *bla*_CMY-4_	IntI1	Omp C, Omp A
I8	Jan, 2009	*Klebsiella pneumoniae*	>256	2	0.19	8	48	>32	8	1	*bla*_SHV-11,_ *bla*_TEM-1,_ *bla*_OXA-1,_ *bla*_CTXM-15_	IntI1	Ompk36, Omp A
I9	Jul, 2009	*Klebsiella pneumoniae*	>256	32	0.125	8	128	>32	1	0.38	*bla*_SHV-1,_ *bla*_TEM-1,_ *bla*_OXA-1,_ *bla*_CTXM-15_	IntI1	Ompk36, Omp A
I10	Aug, 2009	*Klebsiella pneumoniae*	>256	1	0.125	2	>1024	>32	1	0.25	*bla*_SHV-1,_ *bla*_TEM-1,_ *bla*_OXA-1,_ *bla*_CTXM-15_		Ompk36, Omp A
I11	Dec, 2009	*Klebsiella pneumoniae*	>256	0.5	0.38	2	0.75	0.75	0.75	0.5	*bla*_SHV-1_	IntI1	Ompk36, Omp A

CT: Cefotaxime, ETP: Ertapenem, MP: Meropenem, AK: Amikacin, GM: Gentamicin, CI: Ciprofloxacin, TGC: Tigecycline, CL: Colistin; IntI1: class 1 integron; Omp: Outer membrane protein.

^#^ ND: Not Determined

**Table 4:** Antibiotic susceptibility and molecular characterization of NDM-1- harbouring Enterobacteriaceae along with clinical features of the neonates harbouring the same isolates in their blood specimens.

Patient no./Organism(isolate no.)	Sex	InbornOrOutborn	Birthweight	GestationalAge^$^	ModeOfdelivery	ventilation	Prescribedantibiotics	Outcome	MIC Values (mg/L)								Geneticdeterminants	Integron	Porins
									CT	ETP	MP	AK	GM	CI	TGC	CL			
P1/ *Escherichia coli* (E1)	M	Inborn	LBW	preterm	LUCS	No	PipTaz/Amika	discharge	>256	>32	>32	>256	>1024	>32	0.25	1	*bla*_TEM-1,_ *bla*_CTXM_-_15_,*bla*_CMY-6_, *rmt C*, *aac(6’)-Ib*, *ble*_MBL_	IntI1	Omp C, Omp A
P2/ *Escherichia coli* (E2)	M	Inborn	VLBW	preterm	LUCS	Yes	PipTaz/Amika	discharge	>256	>32	8	>256	>1024	>32	0.25	0.5	*bla*_TEM-1_, *bla*_OXA-1_, *bla*_CMY-42_, *arm A*, *aac(6’)-Ib-cr*, *ble*_MBL_		Omp C, Omp F, Omp A
P3/ *Klebsiella pneumoniae* (K1)	M	Outborn	NW	term	NVD	Yes	colistin	discharge	>256	>32	32	>256	>1024	>32	0.75	0.25	*bla*_SHV-11_, *aac(6’)-Ib*, *ble*_MBL_	IntI1	Ompk36, Omp A
P4/ *Klebsiella pneumoniae* (K2)	M	Outborn	LBW	preterm	LUCS	No	ofloxacin	discharge	>256	>32	24	>256	>1024	3	1.5	0.38	*bla*_SHV-167_, *bla*_CTXM_-_15_,*arm A*, *aac(6’)-Ib-cr*, *ble*_MBL_	IntI1	Ompk36, Omp A
P5/ *Klebsiella pneumoniae* (K3)	M	Outborn	LBW	preterm	NVD	Yes	ofloxacin	discharge	>256	12	4	>256	>1024	>32	1	0.75	*bla*_TEM-1_, *bla*_OXA-1_, *bla*_CTXM_-_15_, *aac(6’)-Ib*, *ble*_MBL_	IntI1	Ompk36, Omp A
P6/ *Klebsiella pneumoniae* (K4)	M	Outborn	LBW	term	LUCS	Yes	colistin	discharge	>256	>32	32	>256	>1024	>32	1	0.38	*bla*_TEM-1_, *bla*_OXA-1_, *bla*_CTXM_-_15_, *aac(6’)-Ib*, *ble*_MBL_	IntI1	Ompk36, Omp A
P7/ *Escherichia coli* (E3)	M	Inborn	LBW	preterm	NVD	No	meropenem	death	>256	>32	16	>256	>1024	>32	0.25	0.38	*bla*_TEM-1_, *bla*_CTXM_-_15_, *rmt B*, *ble*_MBL_	IntI1	Omp C, Omp F, Omp A
P8/ *Klebsiella pneumoniae* (K5)	F	Outborn	VLBW	preterm	LUCS	Yes	meropenem	discharge	>256	>32	32	>256	>1024	>32	0.38	0.75	*bla*_SHV-11_, *bla*_OXA-1_, *bla*_CTXM_-_15_, *aac(6’)-Ib*, *ble*_MBL_	IntI1	Ompk36, Omp A
P9/ *Enterobacter cloacae* (EC1)	M	Outborn	LBW	preterm	NVD	Yes	colistin/ofloxacin	LAMA	>256	>32	>32	>256	>1024	4	0.25	1.5	*bla*_OXA-1_, *bla*_CTXM_-_15_, *bla*_ACT-16_, *rmt C*, *aac(6’)-Ib*, *ble*_MBL_	IntI1	Omp C, Omp A
P10/ *Enterobacter cloacae* (EC2)*	M	Outborn	VLBW	preterm	NVD	Yes	colistin	discharge	>256	8	6	>256	>1024	2	0.5	0.5	*bla*_TEM-1_, *bla*_OXA-1_, *bla*_CTXM_-_15_, *rmt B*, *aac(6’)-Ib-cr*, *ble*_MBL_	IntI1	Omp C, Omp A
P10/ *Escherichia coli* (E4)*	M	Outborn	VLBW	preterm	NVD	Yes	colistin	discharge	>256	>32	>32	>256	>1024	>32	0.125	0.75	*bla*_TEM-1_, *bla*_CTXM_-_15_, *bla*_CMY-42_, *rmt B*, *ble*_MBL_	IntI1	Omp C, Omp F, Omp A
P11/ *Escherichia coli* (E5)	M	Inborn	LBW	term	LUCS	No	ofloxacin	discharge	>256	>32	32	>256	>1024	>32	0.38	1.5	*bla*_CTXM_-_15_, *rmt B*, *aac(6’)-Ib-cr*, *ble*_MBL_	IntI1	Omp C, Omp F, Omp A
P12/ *Klebsiella pneumoniae* (K6)**	No clinical data available								>256	12	4	>256	>1024	8	0.75	1	*bla*_TEM-1,_ *bla*_OXA-1_, *bla*_CTXM_-_15_,*rmt B*,*aac(6’)-Ib*,*ble*_MBL_	IntI1	Ompk36,Omp A
P13/ *Enterobacter cloacae* (EC3)	M	Outborn	VLBW	preterm	NVD	Yes	PipTaz/Amika	death	>256	>32	32	>256	>1024	8	1.5	1	*bla*_TEM-1_, *bla*_CTXM_-_15_, *aac(6’)-Ib*, *ble*_MBL_	IntI1	Omp C, Omp A
P14/ *Escherichia coli* (E6)	M	Outborn	VLBW	preterm	NVD	No	ofloxacin	discharge	>256	>32	1.5	>256	>1024	>32	0.094	0.75	*bla*_TEM-1_, *bla*_OXA-1_, *bla*_CTXM_-_15_, *bla*_CMY-42,_ *rmt B*, *aac(6’)-Ib*	IntI1	Omp C, Omp F, Omp A

M: male; F: female; LBW: low birth weight (<2500 gm); VLBW: very low birth weight (<1500 gm); NW: normal weight (>2500 gm); NVD: normal vaginal delivery; LUCS: low uterine caesarean delivery; Omp: Outer membrane protein; LAMA: left against medical advice.

CT: Cefotaxime; ETP: Ertapenem; MP: Meropenem; AK: Amikacin; GM: Gentamicin; CI: Ciprofloxacin; TGC: Tigecycline; CL: Colistin; PipTaz: Piperacillin/Tazobactam; Amika: Amikacin

^$^ Gestational age <37 weeks is considered as preterm.

*Two different Enterobacteriaceae isolates (1 *E*. *coli* and 1 *E*. *cloacae*) were isolated from blood of a single patient.

**No clinical data was available for this patient
