# Supplementary figures and images for: A Five-Year Experience of Carbapenem Resistance in Enterobacteriaceae Causing Neonatal Septicaemia: Predominance of NDM-1
Source: PLoS One. 2014 Nov 18;9(11):e112101. doi: 10.1371/journal.pone.0112101 (PMC4236051; doi:10.1371/journal.pone.0112101)

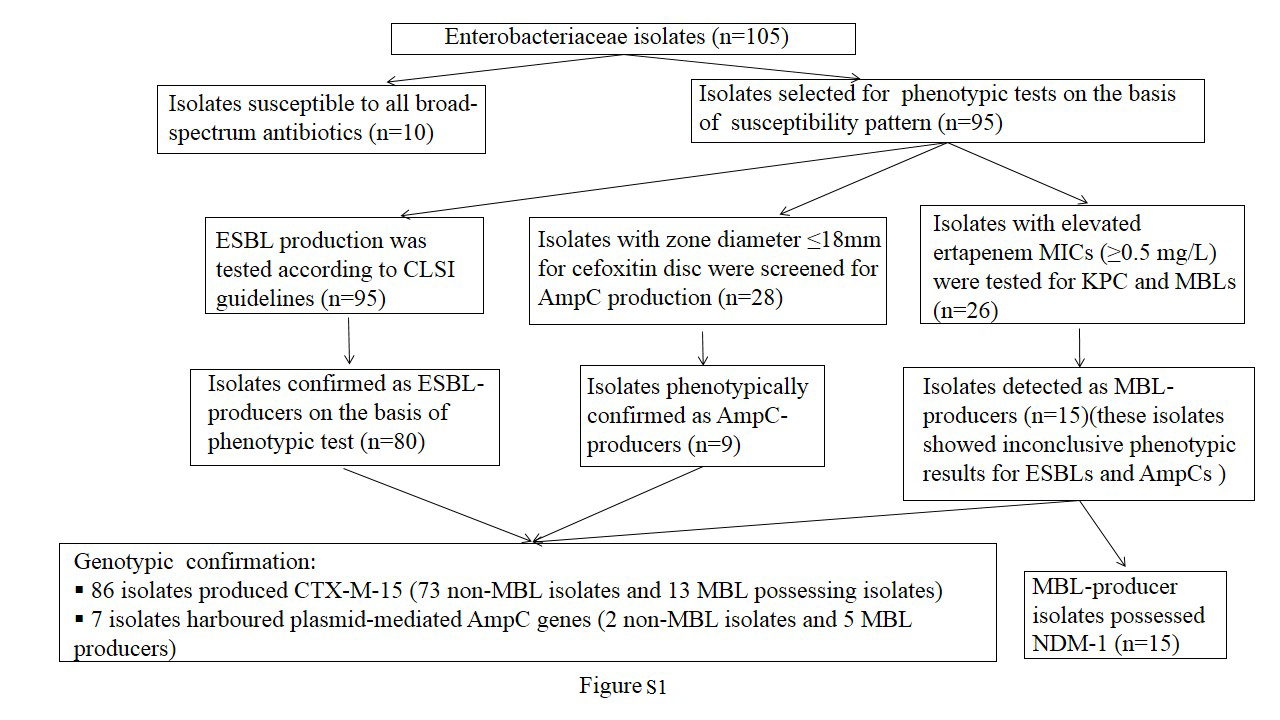

Supplement: Figure S1 — Schematic representation of the molecular analysis of Enterobacteriaceae isolates enrolled in this study. (TIF) [file pone.0112101.s001.tif]
