# Supplementary material for: A Five-Year Experience of Carbapenem Resistance in Enterobacteriaceae Causing Neonatal Septicaemia: Predominance of NDM-1
Source: PLoS One. 2014 Nov 18;9(11):e112101. doi: 10.1371/journal.pone.0112101 (PMC4236051; doi:10.1371/journal.pone.0112101)
Supplement: Table S1 — Susceptibility patterns of the ertapenem susceptible and non-susceptible Enterobacteriaceae isolates for 4 broad spectrum antibiotics. (DOCX) [file pone.0112101.s003.docx]

Table S1. Susceptibility patterns of the ertapenem susceptible and non-susceptible Enterobacteriaceae isolates for 4 broad spectrum antibiotics

|  | Number of isolates (%)^#^ | |
| --- | --- | --- |
|  | ertapenem susceptible (n=79) | ertapenem non-susceptible (n=26) |
| Resistance to Cefotaxime | 62 (78%) | 26 (100%) |
| Resistance to Amikacin | 29 (37%) | 22 (85%) |
| Resistance to Gentamicin | 54 (68%) | 24 (92%) |
| Resistance to Tigecycline | 0 (0%) | 1 (4%) |

^#^ susceptibility was determined according to CLSI-2013 MIC interpretative criteria
