# Supplementary material for: A Five-Year Experience of Carbapenem Resistance in Enterobacteriaceae Causing Neonatal Septicaemia: Predominance of NDM-1
Source: PLoS One. 2014 Nov 18;9(11):e112101. doi: 10.1371/journal.pone.0112101 (PMC4236051; doi:10.1371/journal.pone.0112101)
Supplement: Table S2 — Microbiological and molecular characterization of 105 Enterobacteriaceae isolates included in this study. (DOCX) [file pone.0112101.s004.docx]

| Isolate no. | organism  Table S2. Microbiological and molecular characterization of 105 Enterobacteriaceae isolates included in this study | Year of isolation | MIC values (mg/L) | | | | | | ESBLs, AmpCs, MBLs, 16S rRNA methylases | Integrons |
| --- | --- | --- | --- | --- | --- | --- | --- | --- | --- | --- |
|  |  |  | CT | AK | GM | ETP | MP | TGC |  |  |
| S1 | *E.coli* | 2007 | 0.047 | 4 | 0.5 | 0.008 | 0.032 | 0.19 |  |  |
| S2 | *K. pneumoniae* | 2007 | >32 | ≥256 | ≥1024 | 0.25 | 0.094 | 0.25 | SHV, TEM, OXA-1, CTX-M, Arm A | IntI1 |
| S3 | *K. pneumoniae* | 2007 | >32 | 6 | 0.75 | 0.032 | 0.047 | 0.25 | OXA-1, CTX-M |  |
| S4 | *K. pneumoniae* | 2007 | 0.094 | 12 | 64 | 0.064 | 0.094 | 0.5 | TEM, OXA-1 |  |
| S5 | *E.coli* | 2007 | 0.047 | 2 | 1 | 0.004 | 0.032 | 0.094 |  |  |
| S6 | *E.coli* | 2007 | >32 | 8 | 128 | 0.125 | 0.032 | 0.19 | OXA-1, CTX-M |  |
| S7 | *K. pneumoniae* | 2007 | >32 | ≥256 | ≥1024 | 0.25 | 0.047 | 0.38 | SHV, TEM, CTX-M, Arm A | IntI1 |
| S8 | *K. pneumoniae* | 2007 | 0.125 | 3 | 0.75 | 0.016 | 0.064 | 0.5 |  |  |
| S9 | *K. pneumoniae* | 2007 | >32 | 24 | 3 | 0.125 | 0.064 | 0.25 | TEM, OXA-1, CTX-M |  |
| S10 | *K. pneumoniae* | 2007 | >32 | 12 | 92 | 0.125 | 0.047 | 0.25 | SHV, TEM, OXA-1, CTX-M | IntI1 |
| I1 | *K. pneumoniae* | 2007 | >32 | 8 | 96 | 0.5 | 0.094 | 0.25 | SHV, TEM, OXA-1, CTX-M |  |
| S11 | *K. pneumoniae* | 2007 | >32 | 8 | 48 | 0.032 | 0.064 | 0.25 | SHV, TEM, OXA-1, CTX-M |  |
| S12 | *K. pneumoniae* | 2007 | >32 | 16 | 96 | 0.064 | 0.064 | 0.5 | SHV, TEM, OXA-1, CTX-M | IntI1 |
| S13 | *K. pneumoniae* | 2007 | >32 | 4 | 96 | 0.064 | 0.064 | 0.38 | SHV, TEM, CTX-M |  |
| S14 | *K. pneumoniae* | 2007 | 0.094 | 4 | 0.5 | 0.016 | 0.047 | 0.19 |  |  |
| S15 | *K. pneumoniae* | 2007 | >32 | ≥256 | ≥1024 | 0.25 | 0.064 | 0.5 | SHV, TEM, CTX-M |  |
| S16 | *K. pneumoniae* | 2007 | >32 | ≥256 | ≥1024 | 0.25 | 0.047 | 0.25 | SHV, TEM, CTX-M |  |
| S17 | *K. pneumoniae* | 2007 | >32 | ≥256 | ≥1024 | 0.25 | 0.064 | 0.5 | SHV, TEM, CTX-M | IntI1 |
| S18 | *K. pneumoniae* | 2007 | 192 | 6 | 64 | 0.064 | 0.19 | 0.5 | SHV, TEM, OXA-1, CTX-M | IntI1 |
| S19 | *K. pneumoniae* | 2007 | >32 | ≥256 | ≥1024 | 0.25 | 2 | 0.5 | TEM, CTX-M |  |
| S20 | *K. pneumoniae* | 2007 | >32 | ≥256 | ≥1024 | 0.25 | 0.064 | 0.5 | SHV, TEM, CTX-M, Arm A | IntI1 |
| I2 | *K. pneumoniae* | 2007 | >32 | 24 | 3 | 0.5 | 0.094 | 0.5 | SHV, TEM, OXA-1, CTX-M | IntI1 |
| I3 | *E.coli* | 2007 | >32 | 16 | 128 | 0.5 | 0.19 | 0.064 | OXA-1, CTX-M, Rmt B |  |
| S21 | *K. pneumoniae* | 2007 | >32 | ≥256 | ≥1024 | 0.064 | 0.19 | 0.75 | SHV, TEM, OXA-1, CTX-M, Arm A | IntI1 |
| I4 | *E. cloacae* | 2007 | ND^$^ | ≥256 | ≥1024 | 0.5 | 0.19 | ND | TEM, OXA-1, CTX-M |  |
| I5 | *E. cloacae* | 2008 | ≥256 | 16 | ≥1024 | 24 | .25 | 0.125 | TEM, OXA-1, CTX-M, ACT-7 |  |
| S22 | *E.coli* | 2008 | 1.5 | 3 | 0.50 | 0.008 | 0.016 | 0.064 | SHV, TEM |  |
| S23 | *E.coli* | 2008 | 1.5 | 2 | 0.25 | 0.064 | 0.125 | 0.047 | SHV, TEM, CTXM | IntI1 |
| S24 | *E.coli* | 2008 | 2 | 2 | 0.38 | 0.008 | 0.016 | 0.047 |  |  |
| S25 | *E.coli* | 2008 | ≥32 | 2 | 0.50 | 0.008 | 0.094 | 0.064 | TEM, OXA-1 |  |
| S26 | *E.coli* | 2008 | ≥256 | 12 | 24 | 0.5 | 0.047 | 0.064 | TEM, OXA-1, CTX-M | IntI1 |
| S27 | *E.coli* | 2008 | ≥256 | 2 | 16 | 0.064 | 0.094 | 0.094 |  |  |
| S28 | *E.coli* | 2008 | ≥256 | 8 | 1024 | 0.064 | 0.064 | 1 | SHV, TEM, OXA-1, CTXM | IntI1 |
| S29 | *K. pneumoniae* | 2008 | ≥32 | 12 | 64 | 0.064 | 0.047 | 3 | TEM, OXA-1, CTX-M | IntI1 |
| S30 | *K. pneumoniae* | 2008 | ≥256 | 8 | 96 | 0.032 | 0.094 | 1.5 | SHV, TEM, CTX-M | IntI1 |
| S31 | *K. pneumoniae* | 2008 | ≥32 | 6 | 96 | 0.032 | 0.094 | 1 | TEM, OXA-1 | IntI1 |
| S32 | *K. pneumoniae* | 2008 | 0.064 | 6 | 32 | 0.012 | 0.094 | 1 |  |  |
| S33 | *K. pneumoniae* | 2008 | ≥256 | 12 | 64 | 0.032 | 0.094 | 1.5 | TEM |  |
| S34 | *K. pneumoniae* | 2008 | > 32 | 4 | 16 | 0.032 | 0.094 | 1.5 | TEM, OXA-1, CTX-M | IntI1 |
| S35 | *K. pneumoniae* | 2008 | ≥32 | 6 | 64 | 0.032 | 0.094 | 0.75 | TEM, OXA-1, CTX-M | IntI1 |
| S36 | *K. pneumoniae* | 2008 | 256 | 24 | ≥1024 | 0.125 | 0.125 | 1.5 | SHV, TEM, CTX-M | IntI1 |
| S37 | *K. pneumoniae* | 2008 | ≥256 | ≥256 | ≥1024 | 0.38 | 0.19 | 1 | TEM, OXA-1, CTX-M |  |
| S38 | *K. pneumoniae* | 2008 | ≥256 | ≥256 | 64 | 0.125 | 0.094 | 1 | TEM, CTX-M |  |
| S39 | *K. pneumoniae* | 2008 | ≥256 | ≥256 | ≥1024 | 0.25 | 0.125 | 0.25 | TEM, OXA-1, CTX-M |  |
| S40 | *E. amnigenus* | 2008 | 32 | 6 | 0.38 | 0.125 | 0.047 | 0.50 |  |  |
| I7 | *E.coli* | 2008 | ≥256 | ≥256 | ≥1024 | ≥32 | MIC:1 | 0.064 | TEM, OXA-1, CTX-M, CMY-4 | IntI1 |
| E1 | *E.coli* | 2008 | ≥256 | ≥256 | ≥1024 | ≥32 | ≥32 | 0.25 | TEM, CTX-M, CMY-6, NDM-1 | IntI1 |
| I6 | *K. pneumoniae* | 2008 | ≥256 | 12 | 162 | 0.5 | 0.047 | 0.5 | TEM, OXA-1, CTX-M | IntI1 |
| S41 | *K. pneumoniae* | 2009 | ≥256 | 3 | 0.5 | 0.25 | 0.19 | 0.72 | SHV, TEM, CTX-M | IntI1 |
| I8 | *K. pneumoniae* | 2009 | ≥32 | 8 | 48 | 2 | 0.19 | 8 | SHV, TEM, OXA-1, CTXM | IntI1 |
| S42 | *E.coli* | 2009 | ≥32 | 2 | 0.38 | 0.032 | 0.19 | 0.064 | CTX-M |  |
| S43 | *K. pneumoniae* | 2009 | 96 | 8 | 64 | 0.125 | 0.125 | 0.5 | SHV, TEM, CTX-M | IntI1 |
| S44 | *K. pneumoniae* | 2009 | ≥32 | 6 | 192 | 0.25 | 0.125 | 1.5 | SHV, TEM, OXA-1, CTXM | IntI1 |
| E2 | *E.coli* | 2009 | ≥256 | ≥256 | ≥1024 | ≥32 | 8 | 0.25 | TEM, OXA, CMY-42, NDM-1, Arm A |  |
| S45 | *K. pneumoniae* | 2009 | ≥256 | 3 | 64 | 0.125 | 0.25 | 0.5 | SHV, TEM, CTX-M | IntI1 |
| I9 | *K. pneumoniae* | 2009 | ≥256 | 8 | 128 | 32 | 0.125 | 1 | SHV, TEM, OXA-1, CTXM | IntI1 |
| S46 | *E. sakazakii* | 2009 | 0.38 | 3 | 2 | 0.032 | 0.064 | 0.38 |  |  |
| I10 | *K. pneumoniae* | 2009 | ≥256 | 2 | ≥1024 | 1 | 0.125 | 1 | SHV, TEM, OXA-1, CTXM |  |
| S47 | *K. pneumoniae* | 2009 | 0.032 | 1.5 | 0.38 | 0.032 | 0.047 | 0.19 | SHV | IntI1 |
| S48 | *E. cloacae* | 2009 | ≥256 | 32 | ≥1024 | 0.25 | 0.125 | 1 | TEM, OXA-1, CTX-M | IntI1 |
| S49 | *K. pneumoniae* | 2009 | 48 | 6 | 24 | 0.032 | 0.047 | 0.19 | SHV, TEM, OXA-1, CTXM | IntI1 |
| I11 | *K. pneumoniae* | 2009 | 64 | 2 | 0.75 | 0.5 | 0.38 | 0.75 | SHV | IntI1 |
| S50 | *K. pneumoniae* | 2010 | ≥256 | ≥256 | ≥1024 | 0.25 | 0.047 | 1 | SHV, TEM, CTX-M | IntI1 |
| K1 | *K. pneumoniae* | 2010 | ≥256 | ≥256 | ≥1024 | ≥32 | 32 | 0.75 | SHV, NDM-1 | IntI1 |
| K2 | *K. pneumoniae* | 2010 | ≥256 | ≥256 | ≥1024 | ≥32 | 24 | 1.5 | SHV, CTX-M, NDM-1, Arm A | IntI1 |
| S51 | *K. pneumoniae* | 2010 | ≥256 | 24 | 1.5 | 0.064 | 0.25 | 0.25 | SHV, TEM, CTX-M | IntI1 |
| S52 | *K. pneumoniae* | 2010 | ≥256 | 16 | 1.5 | 0.125 | 0.064 | 0.5 | SHV, TEM, CTX-M | IntI1 |
| S53 | *K. pneumoniae* | 2010 | ≥256 | 16 | 1.5 | 0.032 | 0.094 | 0.19 | SHV, TEM, CTX-M | IntI1 |
| S54 | *K. pneumoniae* | 2010 | ≥256 | 24 | ≥1024 | 0.064 | 0.094 | 0.5 | CTX-M | IntI1 |
| S55 | *K. pneumoniae* | 2010 | ≥256 | 6 | 128 | 0.016 | 0.047 | 0.5 | SHV, TEM, CTX-M |  |
| S56 | *E. cloacae* | 2010 | ≥256 | 24 | 2 | 0.25 | 0.032 | 0.25 | SHV, TEM, CTX-M | IntI1 |
| S57 | *E.coli* | 2010 | ≥32 | 16 | 128 | 0.125 | 0.125 | 0.19 | SHV, TEM, CTX-M | IntI1 |
| S58 | *K. pneumoniae* | 2010 | ≥256 | 16 | 0.75 | 0.125 | 0.125 | 0.125 | SHV, TEM, CTX-M | IntI1 |
| K3 | *K. pneumoniae* | 2010 | ≥256 | ≥256 | ≥1024 | 12 | 4 | 1 | TEM, OXA-1, CTX-M, NDM-1 | IntI1 |
| K4 | *K. pneumoniae* | 2010 | ≥256 | ≥256 | ≥1024 | ≥32 | 32 | 1 | TEM, OXA-1, CTX-M, NDM-1 | IntI1 |
| E3 | *E.coli* | 2010 | ≥256 | ≥256 | ≥1024 | ≥32 | 16 | 0.25 | TEM, CTX-M, NDM-1 | IntI1 |
| S59 | *E.coli* | 2010 | ≥256 | 24 | ≥1024 | 0.064 | 0.032 | 0.5 | CTX-M |  |
| K5 | *K. pneumoniae* | 2010 | ≥256 | ≥256 | ≥1024 | ≥32 | 32 | 0.38 | SHV, OXA-1, CTX-M, NDM-1 | IntI1 |
| S60 | *K. pneumoniae* | 2010 | ≥256 | 16 | 48 | 0.125 | 0.125 | 0.38 | TEM, CTX-M | IntI1 |
| S61 | *K. pneumoniae* | 2010 | ≥256 | 8 | 48 | 0.064 | 0.047 | 1 | OXA-1, CTX-M, Rmt C | IntI1 |
| EC1 | *E. cloacae* | 2010 | ≥256 | ≥256 | ≥1024 | ≥32 | ≥32 | 0.25 | TEM, OXA-1, CTX-M, ACT-16, NDM-1 | IntI1 |
| S62 | *E. cloacae* | 2010 | ≥256 | ≥256 | ≥1024 | 0.25 | 0.125 | 0.5 | TEM, OXA-1, CTX-M | IntI1 |
| S63 | *K. pneumoniae* | 2010 | ≥256 | 6 | 192 | 0.125 | 0.094 | 0.25 | SHV, TEM, OXA-1, CTXM | IntI1 |
| S64 | *E.coli* | 2010 | 1.5 | 6 | 64 | 0.016 | 0.032 | 0.047 | TEM, CTX-M |  |
| S65 | *E.coli* | 2011 | 0.064 | 12 | 32 | 0.064 | 0.125 | 0.25 | SHV, TEM, CTX-M | IntI1 |
| S66 | *K. pneumoniae* | 2011 | 0.064 | 8 | 96 | 0.064 | 0.064 | 0.25 | SHV, TEM, OXA-1, CTXM | IntI1 |
| S67 | *E.coli* | 2011 | 0.125 | 2 | 0.125 | 0.125 | 0.047 | 0.064 | CTX-M |  |
| S68 | *E.coli* | 2011 | 0.006 | 2 | 0.5 | 0.006 | 0.032 | 0.064 | CTX-M | IntI1 |
| EC2 | *E. cloacae* | 2011 | ≥32 | ≥256 | ≥1024 | 8 | 6 | 0.5 | TEM, OXA-1, CTX-M, NDM-1, Rmt B | IntI1 |
| E4 | *E.coli* | 2011 | ≥32 | ≥256 | ≥1024 | ≥32 | ≥32 | 0.125 | TEM, CTX-M, CMY-42, NDM-1, Rmt B | IntI1 |
| S69 | *E.coli* | 2011 | 0.064 | 2 | 0.25 | 0.008 | 0.032 | 0.094 | SHV, TEM, OXA-1 | IntI1 |
| S70 | *K. pneumoniae* | 2011 | ≥32 | 16 | 128 | 0.016 | 0.064 | 0.38 | CTX-M | IntI1 |
| S71 | *K. pneumoniae* | 2011 | ≥32 | 8 | 96 | 0.094 | 0.094 | 0.38 |  |  |
| S72 | *K. pneumoniae* | 2011 | ≥32 | ≥256 | ≥1024 | 0.19 | 0.094 | 0.19 | SHV, TEM, OXA-1, CTXM | IntI1 |
| E5 | *E.coli* | 2011 | ≥32 | ≥256 | ≥1024 | ≥32 | 32 | 0.38 | CTX-M, Rmt B, NDM-1 | IntI1 |
| S73 | *K. pneumoniae* | 2011 | ≥32 | 4 | 132 | 0.125 | 0.094 | 1 | SHV, OXA-1, CTX-M | IntI1 |
| S74 | *K. pneumoniae* | 2011 | 256 | 24 | 96 | 0.25 | 0.064 | 2 | CTX-M | IntI1 |
| S75 | *K. pneumoniae* | 2011 | ≥32 | 12 | 96 | 0.25 | 0.064 | 1.5 | CTX-M | IntI1 |
| K6 | *K. pneumoniae* | 2011 | ≥256 | ≥256 | ≥1024 | 12 | 4 | 0.75 | TEM, OXA-1, CTX-M, NDM-1, Rmt B | IntI1 |
| EC3 | *E. cloacae* | 2011 | ≥32 | ≥256 | ≥1024 | ≥32 | 32 | 1.5 | TEM, CTX-M | IntI1 |
| E6 | *E.coli* | 2011 | ≥256 | ≥256 | ≥1024 | ≥32 | 1.5 | 0.094 | TEM, OXA-1, CTX-M, CMY-42, NDM-1, Rmt B | IntI1 |
| S76 | *K. pneumoniae* | 2011 | ≥32 | 6 | 24 | 0.125 | 0.064 | 0.38 | CTX-M | IntI1 |
| S77 | *E.coli* | 2011 | ≥32 | 5 | 0.5 | 0.016 | 0.047 | 1 | CTX-M | IntI1 |
| S78 | *K. pneumoniae* | 2011 | ≥32 | 6 | 0.75 | 0.125 | 0.094 | 1 | SHV, TEM, OXA-1, CTXM | IntI1 |
| S79 | *K. pneumoniae* | 2011 | ≥32 | 6 | 48 | 0.064 | 0.094 | 0.25 | CTX-M | IntI1 |

^$^ND: Not Determined

CT: Cefotaxime, ETP: Ertapenem, MP: Meropenem, AK: Amikacin, GM: Gentamicin, TGC: Tigecycline, IntI1: class 1 Integron;
